# Ovarian Carcinosarcoma: Effects of Cytoreductive Status and Platinum-Based Chemotherapy on Survival

**DOI:** 10.1155/2013/490508

**Published:** 2013-05-27

**Authors:** Amelia M. Jernigan, Amanda Nickles Fader, Benjamin Nutter, Peter Rose, Jill H. Tseng, Pedro F. Escobar

**Affiliations:** ^1^Johns Hopkins Hospital, 1800 Orleans Street, Baltimore, MD 21287, USA; ^2^Greater Baltimore Medical Center, 6701 N. Charles Street, Towson, MD 21204, USA; ^3^Cleveland Clinic, 9500 Euclid Avenue, Desk A-81, Cleveland, OH 44195, USA; ^4^HIMA, 100 Luis Muñoz Marín Avenue, Caguas 00725, Puerto Rico

## Abstract

*Objective*. To define survival patterns of women with ovarian carcinosarcoma based on patient, tumor, and treatment characteristics. *Methods/Materials*. A single-institution, retrospective analysis of women diagnosed with ovarian carcinosarcoma from February 1993 to May 2009 was performed. Survival was analyzed with Cox proportional hazards ratios and Kaplan Meier tests. *Results*. Forty-seven cases of primary ovarian carcinosarcoma were identified. Age conveyed an HR 3.28 (95% CI 1.51–7.11, *P* = 0.003) for death. Compared to Stages I-II, Stage III carried an HR for death of 4.75 (95% CI 1.16–19.4, *P* = 0.03) and Stage IV disease an HR of 9.13 (95% CI 1.76–47.45, *P* = 0.009). Compared to those with microscopic residual, women with >1 cm diameter of residual disease after primary cytoreductive surgery had an HR for death of 4.71 (95% CI 1.84–12.09, *P* = 0.001). At analysis, 59.1% of those who received platinum-based chemotherapy were alive, compared to 23.1% of those who received nonplatinum-based chemotherapy (*P* = 0.08). *Conclusions*. Age, stage, and cytoreduction to no gross residual disease are associated with improved survival in women with ovarian carcinosarcoma. Complete surgical cytoreduction should be the goal of surgical management when possible, but the ideal adjuvant treatment regimen remains unclear.

## 1. Introduction

Ovarian cancer is the second most common gynecologic cancer, diagnosed in more than 20,000 women annually in the USA, and it is one of the most lethal female malignancies [1]. Ovarian carcinosarcoma is a rare histologic subtype of ovarian cancer that is diagnosed in 1%–4% of all ovarian cancer survivors [2–4]. It is a biologically aggressive tumor that is routinely widely metastatic at the time of initial presentation. Reports from SEER and other databases demonstrate that when matched by stage ovarian carcinosarcoma has a poorer prognosis and survival than epithelial ovarian cancer [4–6]. There is no uniform agreement regarding the optimal treatment of ovarian carcinosarcoma. Most published data are limited to retrospective reviews, and because of the rarity of the disease, few institutions are able to accrue a sufficient number of patients for prospective studies. Some studies suggest that an optimal surgical cytoreductive effort at the time of primary surgery and adjuvant chemotherapy may be associated with improved overall survival [2, 7–9]. Our assumptions regarding the best surgical treatments and adjuvant therapies for this disease are extrapolated from studies of patients with epithelial ovarian carcinoma or uterine carcinosarcoma. 

There are limited reports in the literature to date that define the prognostic factors and optimal treatment strategies associated with survival in women with ovarian carcinosarcoma. The purpose of this study was to define the clinicopathological variables of outcome and survival patterns of women with primary ovarian carcinosarcoma.

## 2. Methods

An institutional review board-approved, single-institution retrospective analysis of women with a primary diagnosis of ovarian or fallopian tube carcinosarcoma was performed at the Cleveland Clinic, Cleveland, OH. The study period was from 1993 to 2009. We began collecting patients when reliable electronic records were available and attempted to allow for at least 36 months of followup prior to data analysis. All pathology obtained had been reviewed by a gynecologic pathologist at this center. Study subjects were excluded if they had a primary diagnosis of uterine, cervical, or other nonovarian carcinosarcoma. If the tumor involved the endometrial cavity or cervix, it was considered most likely a uterine carcinosarcoma and was excluded.

 Variables recorded included age at diagnosis, preoperative CA 125 (units/mL), stage, surgical procedures performed, adjuvant therapies administered, date of last followup, and date of death, when applicable. As it is commonplace for our surgeons to detail the centimeters of residual disease in their operative report after primary cytoreductive surgery, we were able to record their residual disease status to the centimeter in all 47 subjects. Residual disease status after primary cytoreductive surgery was defined as microscopic residual (no gross residual disease), 0.1–1.0 cm maximal diameter residual disease, or >1.0 cm maximal diameter residual disease. For all study subjects with a recorded death, this was confirmed by performing a social security death index search. Survival time was defined from the date of diagnosis to the date of last followup or date of death via the social security death index or medical record. 

 Data were described using median and ranges for numeric variables and frequency and percentage for categorical variables. Univariable comparisons were made using Wilcoxon's signed rank test for numeric factors. Categorical factors were compared using a chi-square test unless Fisher's exact test was deemed more appropriate. For continuous variables, such as age, hazard ratios are expressed as the increase in hazard relative to a change from the 1st to the 3rd quartile of the variable's distribution. Hazard ratios are calculated from Cox proportional hazard models; Kaplan-Meier survival curves are provided to illustrate comparisons between groups. Analyses were conducted in R 2.12.2 (R Foundation for Statistical Computing) with significance determined by a *P* value less than or equal to 0.05.

## 3. Results

 We identified 47 patients with primary ovarian carcinosarcoma.  Table 1 describes characteristics of subjects and their tumor and treatment profiles. The median age was 66 (range 20–88 years). The majority had advanced stage disease (75% of those in whom we had accurate staging data), underwent an optimal primary cytoreductive procedure (70.2%), and received platinum-based adjuvant therapy (57.9% of those in whom we had records of the adjuvant therapy received). 

With regards to surgical treatment, information on the degree of residual disease after initial cytoreductive surgery was available for all 47 patients. Most (70.2%) were optimally cytoreduced to ≤1 centimeter at the end of their procedure, and over half (51.1%) were cytoreduced to microscopic, or no gross residual disease. However, almost one-third (29.8%) were suboptimally cytoreduced, left with more than 1 centimeter of residual disease. Lymphadenectomy was performed in 27.7% of cases, and approximately 22 subjects (46.8%) underwent a staging procedure that included resection of the bowel and/or spleen. 

Initial adjuvant therapy regimens were recorded, with data available for 38 subjects. Amongst these 38, 57.9% received platinum-based chemotherapy regimens such as single agent carboplatin, cyclophosphamide/adriamycin/platinum, carboplatin/paclitaxel, cisplatin/ifosfamide, carboplatin/ifosfamide, and carboplatin/gemcitabine. Nonplatinum-based chemotherapy included doxorubicin, Adriamycin/ifosfamide, and ifosfamide and were administered to 34.2% of these subjects. Three subjects underwent whole pelvic radiation therapy alone. 

On univariate analysis, age (*P* = 0.006), stage (*P* = 0.034), and cytoreductive status (*P* = 0.005) were all significantly associated with overall survival. Specifically advanced age, advanced stage, and residual disease >1 cm after primary cytoreductive surgery were associated with poor survival outcomes. When examining adjuvant treatment associations, there was a trend associated with survival in women who received platinum-based chemotherapy regimens versus non-platinum-based regimens (*P* = 0.08). 

Survival data were available for all 47 subjects. Median follow-up and overall survival time was 25.8 months (range 0.1–213 months). At the time of analysis 21 (44.7%) of subjects were still alive. On multivariable analysis, age, stage, and cytoreductive status were independently associated with survival (Tables 2 and 3). Older patients had a Cox proportional HR for death of 3.28 (95% CI 1.51–7.11, *P* = 0.003). When compared to stages I and II disease, stage III disease had an HR for death of 4.75 (95% CI 1.16–19.4, *P* = 0.03) and stage IV disease had HR of 9.13 (95% CI 1.76–47.45, *P* = 0.009). Women left with ≥1 cm of residual disease had an HR for death of 4.71 (95% CI 1.84–12.09, *P* = 0.001) for death compared to those with no gross residual disease remaining after primary cytoreductive surgery. 

**Figure 1:**
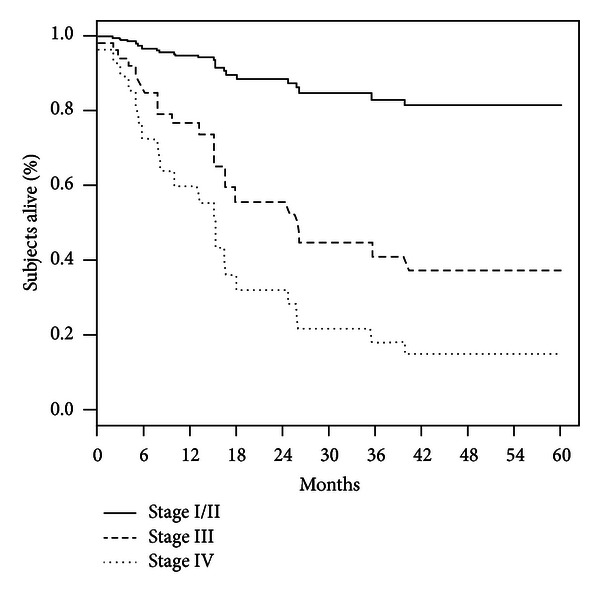
Age-adjusted survival by stage.

**Figure 2:**
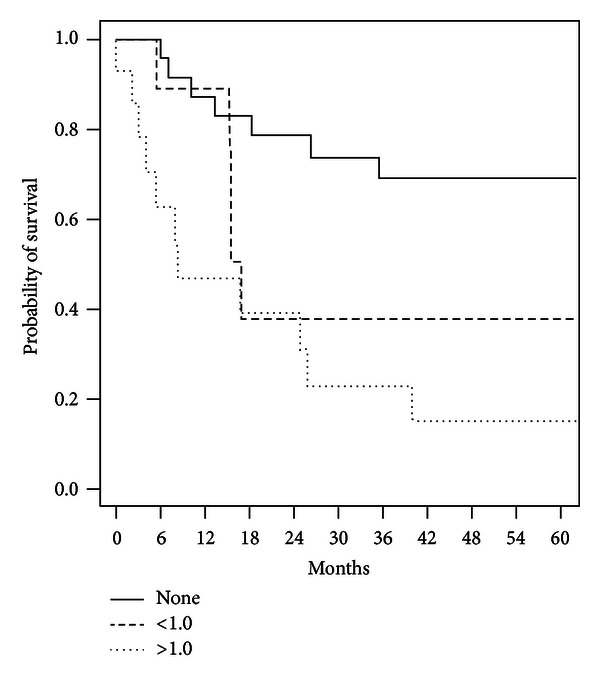
Age-adjusted survival by cytoreductive status.

Finally, Kaplan-Meier curves illustrating age-adjusted survival by stage (Figure 1) and cytoreductive status (Figure 2) are shown and highlight the significant associations of these variables with overall survival. 

## 4. Discussion

To our knowledge, this retrospective study is one of the largest contemporary, single-institution series of ovarian carcinosarcoma in the literature. Our findings corroborate other reports that have demonstrated that ovarian carcinosarcoma is an extremely aggressive subtype of epithelial ovarian cancer that is often advanced at diagnosis [2–4, 10], and that age and stage are associated with overall survival [2, 5, 9, 11, 12]. Furthermore, we showed that extent of primary cytoreductive surgery and residual disease status correlate with outcome, consistent with previous reports. However, most of these older retrospective studies were smaller and utilized an older definition of “optimal cytoreduction” (≤2 centimeters diameter of residual disease remaining) [3, 5, 11–13]. The definition of optimal cytoreduction has since been modified to indicate no larger than 1 centimeter in diameter of residual disease after primary surgery [14]. Our report is amongst the first to examine the relationship between survival and residual disease at this level; in our analysis, enhanced survival outcomes are associated with cytoreduction to no gross residual disease in ovarian carcinosarcoma. 

While the current Gynecologic Oncology Group definition of “optimal cytoreduction” is a surgical effort that leaves no more than 1 centimeter diameter of residual disease, there is increasing evidence that a paradigm shift in favor of cytoreduction to no gross residual disease is warranted [15]. Chang et al. argue that the goal of initial surgical management of epithelial ovarian carcinoma should be cytoreduction to no residual disease [15]. Until recently, this newer proposed definition of “optimal” management has not been applied or evaluated in the setting of ovarian carcinosarcoma, but our data support the concept that no gross residual disease is associated with improved survival outcomes over traditional “optimal” cytoreduction to ≤1 centimeter. In our analysis, even when controlling for age and stage, the association between cytoreduction to no gross residual disease and survival remains. In another large retrospective study of subjects with ovarian carcinosarcoma, Rauh-Hain et al. demonstrated an OS of 47 months for patients with microscopic residual disease, 18 months with macroscopic residual disease ≤1 cm, and 8 months with suboptimal cytoreduction (*P* = 0.02) [6]. Given the rarity of this tumor type, what we know about cytoreduction can be ascertained from small, retrospective studies which cannot conclude a cause and effect relationship between cytoreduction and survival. Nonetheless, recent studies have repeatedly associated aggressive primary surgical outcomes, such as removal of all visible tumor, with better survival [6, 9]. Our results corroborate these recent reports and suggest the potential benefit of an aggressive surgical cytoreductive effort.

However, the determination of the optimal chemotherapy regimen has been elusive. In one of the largest prospective studies of women with ovarian carcinosarcoma, the Gynecologic Oncology Group enrolled 136 patients over a 20-year period and concluded that cisplatin had modest activity (20%) but that further prospective studies of cytotoxic agents would not be feasible because of the rarity of the disease. The authors recommended extrapolating data from the more common variant, uterine carcinosarcomas. Some data is also extrapolated from what we know about epithelial ovarian carcinomas but should be interpreted with caution. Brown et al. reported that women with ovarian carcinosarcoma demonstrate a relatively blunted response to chemotherapy compared to women with epithelial ovarian carcinoma [5].

In ovarian and endometrial carcinosarcomas, ifosfamide [16] and platinum [17–19] have demonstrated efficacy. The carboplatin and paclitaxel combination has been reported with a response rate up to 72% in carcinosarcomas of the reproductive tract and seems to have a more favorable toxicity profile than ifosfamide or cisplatin [11, 13, 20]. Aggressive combinations of anthracylines, alkalating agents, and platinums are active but have significant toxicity, with some reports demonstrating almost two-thirds of the treatment group delaying or changing treatment [21, 22]. Largely due to feasibility issues, a quality head-to-head comparison of the adjuvant therapies has not been performed until now. The Gynecologic Oncology Group is currently enrolling study subjects for GOG 261, a phase III trial of carboplatin and paclitaxel versus ifosfamide and paclitaxel for the treatment of women with primary, Stage IV uterine or ovarian carcinosarcomas. Correlative endpoints of this trial may reveal pathways and molecular signatures that are common to the gynecologic carcinosarcomas that will help to identify novel, targeted therapies to better treat this histologic subtype of ovarian cancer.

In this study, we attempted to determine if platinum-based adjuvant therapy was associated with a survival benefit, but we did not detect a difference. There may be benefit to utilizing platinum-based therapy in this setting, but the small number of patients in this study limited our ability to draw any definitive conclusion. Notably, a trend was demonstrated suggesting that platinum-based therapy may be associated with survival when compared to regimens that are not platinum-based. Chun et al. recently retrospectively demonstrated that women who received paclitaxel/platinum-based combinations experienced a longer progression free interval and overall survival [9]. Furthermore, toxicity and the ability to complete a regimen should be considered. In a recent retrospective series of 81 Swedish cases, Paulsson et al. demonstrated that, compared to those with an incomplete regimen, the 57% of subjects who completed 6 cycles of platinum-based chemotherapy had improved overall survival [10]. 

 Study weaknesses include the retrospective study design and its intrinsic limitations, the small sample size and the heterogeneity in surgical interventions and adjuvant therapies. However, study strengths include that this is one of the largest single-institution series of ovarian carcinoma that identified potentially modifiable prognostic factors for survival. In anticipation of GOG 261, it will be interesting to learn whether the prognostic factors identified in the current study (age, stage, and cytoreductive status) especially cytoreduction to no gross residual disease will be corroborated in the phase III trial results. 

This report demonstrates that complete cytoreduction to no gross residual disease is associated with improved survival outcomes in women with ovarian carcinosarcoma. Given the limited activity of cytotoxic chemotherapy in the treatment of this disease, a maximal surgical effort with the goal of cytoreduction to no gross residual disease should be the goal of initial management whenever possible. The ideal adjuvant treatment regimen is unclear, but a trend was observed favoring platinum-based therapy [15]. We eagerly await the results of GOG 261, to shed some light on this matter.

## Figures and Tables

**Table 1:** Clinicopathologic and treatment characteristics. Statistics are reported in median with ranges and percentages.

Factor	*N*	Statistics
Median age^a^	47	66 (20, 88) years
Median preoperative CA-125^a^	32	117 (16, 986) units/mL
Stage^b^		
I/II	11	23.4%
III	27	57.4%
IV	6	12.9%
Unknown	3	6.4%
Cytoreductive status^b^		
No residual disease	24	51.1%
Less than 1 cm residual disease	9	19.1%
More than 1 cm residual disease	14	29.8%
Lymph node dissection^b^		
No	34	72.3%
Yes	13	27.7%
Adjuvant therapy^b^		
Platinum-based chemotherapy	22	46.8%
Nonplatinum-based chemotherapy	13	27.7%
Radiation therapy	3	6.4%
Unknown	9	19.1%

^a^Range (minimum, maximum); ^b^percentage.

**Table 2:** Hazard ratios for death by age and stage. Univariable and Cox proportional hazard ratios demonstrating the association between advanced age and stage with an increased risk of death.

Factor	Cox proportional hazard ratio	95% confidence interval	*P* value
Age (1st compared to 3rd quartiles, 54.5 versus 73.5 years)	3.28	1.51–7.11	0.003*
Stage III (compared to Stages I and II)	4.75	1.16–19.4	0.03*
Stage IV (compared to Stages I and II)	9.13	1.76–47.45	0.009*

*Statistically significant with *P* < or = 0.05.

**Table 3:** Hazard ratios for death compared to complete cytoreduction to no gross residual disease. Hazard Ratios demonstrating the association between the degree of cytoreduction and survival.

	Amount of residual disease	Univariable hazard ratio	95% confidence interval	*P* value
All stages	≤1 cm	1.77	0.56–5.55	0.33
	>1 cm	4.71	1.84–12.09	0.001*
Stages II and IV	≤1 cm	1.50	0.044–5.13	0.51
	>1 cm	3.41	1.21–9.62	0.02*

*Statistically significant with *P* < or = 0.05.
